# Icariin Induces Synoviolin Expression through NFE2L1 to Protect Neurons from ER Stress-Induced Apoptosis

**DOI:** 10.1371/journal.pone.0119955

**Published:** 2015-03-25

**Authors:** Fei Li, Beixue Gao, Hongxin Dong, Jingshan Shi, Deyu Fang

**Affiliations:** 1 Department of Pharmacology and the Key Laboratory of Basic Pharmacology of Guizhou Province, Zunyi Medical College, Zunyi, China; 2 Department of Pathology, Northwestern University Feinberg School of Medicine, 303 E. Chicago Ave, Chicago, IL, 60611, United States of America; 3 Department of Psychiatry and Behavioral Sciences, Northwestern University Feinberg School of Medicine, 303 E. Chicago Ave, Chicago, IL, 60611, United States of America; University of Pittsburgh School of Medicine, UNITED STATES

## Abstract

By suppressing neuronal apoptosis, Icariin is a potential therapeutic drug for neuronal degenerative diseases. The molecular mechanisms of Icariin anti-apoptotic functions are still largely unclear. In this report, we found that Icariin induces the expression of Synoviolin, an endoplasmic reticulum (ER)-anchoring E3 ubiquitin ligase that functions as a suppressor of ER stress-induced apoptosis. The nuclear factor erythroid 2-related factor 1 (NFE2L1) is responsible for Icariin-mediated Synoviolin gene expression. Mutation of the NFE2L1-binding sites in a distal region of the Synoviolin promoter abolished Icariin-induced Synoviolin promoter activity, and knockdown of NFE2L1 expression prevented Icariin-stimulated Synoviolin expression. More importantly, Icariin protected ER stress-induced apoptosis of PC12 cells in a Synoviolin-dependent manner. Therefore, our study reveals Icariin-induced Synoviolin expression through NFE2L1 as a previously unappreciated molecular mechanism underlying the neuronal protective function of Icariin.

## Introduction

Icariin, the prenyl acetylation of kaempferide 3,7-*O*-diglucoside, is a flavonoid derived from the genus *Epimedium*, a plant known as Horny Goat Weed or *Yin Yang Huo* [1]. Extracts from these plants are used in Chinese herbal medicine to enhance erectile function. Recent studies, including work from our laboratory, have shown that Icariin has a potent neuronal protective activity and prevents neuronal degenerative disease in mouse models [2–20]. Several pathways are likely involved in Icariin-induced neuronal cell proliferation and inhibition of apoptosis [13, 14, 21–27]. We have shown that Icariin inhibits β-amyloid peptide segment 25–35—induced expression of β-secretase in the rat hippocampus [10, 28–30]. Icariin also attenuates β-amyloid–induced neurotoxicity by inhibition of tau protein hyperphosphorylation [9]. More recently, Zhang et al observed that Icariin may prevent corticosterone-induced cell death via activation of the PI3-K/Akt pathway in neuronal cells [4]. In addition to its direct effect on neurons, Icariin inhibits both TAK1/IKK/NF-kappaB and JNK/p38 MAPK pathways in microglial cells to suppress inflammatory cytokine production, thus indirectly protecting neurons from inflammatory injury [7, 13, 16, 31–34]. An elevated ER stress response has been detected in brain tissues from patients with neuronal degenerative diseases, and ER stress-induced cell death has been considered as one of the possible casual factors of neuronal cell death in these diseases [35–42]. However, whether the neural protective activity of Icariin involves suppression of ER stress-induced neuronal cell apoptosis remains to be determined.

Synoviolin, also known as Hrd1, is an ER membrane-spanning protein. Synoviolin was initially identified as a ubiquitin ligase involved in degrading misfolded proteins [43, 44]. Since Hrd1 expression is often upregulated in synovial fibroblasts in patients with rheumatoid arthritis, it was renamed Synoviolin [45]. We recently reported that proinflammatory cytokines, including TNF-α and IL-1β, are responsible for inducing Synoviolin expression in synovial fibroblasts [46]. Synoviolin achieves its anti-apoptotic functions through multiple mechanisms: first, Synoviolin degrades misfolded proteins to suppress ER stress-induced cell death [47]; second, we discovered that Synoviolin ubiquitinates IRE1α (inositol-requiring enzyme 1α), a critical kinase regulating ER stress-induced cell death [48]; and third, Synoviolin has been identified as an E3 ubiquitin ligase that targets p53, implying that Synoviolin may inhibit apoptosis through cytoplasmic p53 degradation [49].

In the current study, we observed that Icariin induces expression of the anti-apoptotic factor Synoviolin at the gene transcriptional level. Further analysis demonstrated that the transcription factor NFE2L1 is responsible for Icariin-induced Synoviolin mRNA transcription. Consequently, Icariin protects ER stress-induced neuronal cell death in a Synoviolin-dependent manner. Thus, our studies identify Synoviolin as a molecular link between Icariin and its neuronal anti-apoptotic activity.

## Materials and Methods

### Cells and reagents

PC12 cells were cultured in D-MEM (Gibco, Grand Island, NY, USA) supplemented with 10% fetal bovine serum, 50 μM β-mercaptoethanol, 100 mM sodium pyruvate, 100 mM HEPES buffer, and 1% penicillin/streptomycin. Specific antibodies against Synoviolin (H7790) and β-Actin were purchased from Sigma (St. Louis, MO, USA) and NFE2L1 (SC-721) from Santa Cruz (Santa Cruz, CA, USA). siRNA specific to Synoviolin and NFE2L1 (SR504532), as well as controls, were from Origene (Rockville, MD, USA).

### Immunoblot analysis

PC12 cells were treated with Icariin at the indicated concentrations and harvested after 24 hours. Cells were lysed in radioimmunoprecipitation (RIPA) buffer containing phosphatase inhibitors and protease inhibitors (Roche Diagnostics, Indianapolis, IN, USA). Cell lysates were boiled with Laemlli’s buffer, then subjected to SDS-PAGE and transferred to nitrocellulose membrane. Membranes were blocked with 5% milk in TBS-T, incubated with the indicated primary antibodies overnight, followed by incubation with HRP-conjugated secondary antibody as described [50]. Immunoblots were developed using enhanced chemiluminescent substrate (Pierce, Rockford, IL, USA) and visualized using the ChemiDoc XRS+ System (BioRad, Hercules, CA, USA).

### Real-time quantitative PCR

Quantification of mRNA expression was performed as described [51]. 10^6^ PC12 cells were lysed in Trizol (Invitrogen, Carlsbad, CA, USA), and RNA isolated per manufacturer’s instructions. 1 μg of isolated RNA was then reversed transcribed using the qScript cDNA synthesis kit (Quanta BioSciences, Gaithersburg, MD, USA). Real-time quantitative PCR (qPCR) was performed in duplicate wells using the iCycler Sequence and SsoFast SYBR Green Supermix (BioRad). Relative expression was normalized to expression of *Actb*.

### Luciferase reporter assay

The Synoviolin promoter region was amplified by PCR and subcloned into the pGL4.10(luc2) vector (Promega, Madison, WI, USA). PC12 cells in 12-well plates were transfected with pRL-TK (Promega) and various pSynoviolin luciferase plasmids as indicated using Lipofectamine transfection reagent (Invitrogen Life Technologies, San Diego, CA, USA). Luciferase assays were performed as previously reported [52]. The pRL-TK plasmid contains the *Renilla reniformis* (sea pansy) luciferase gene under the transcriptional control of the herpesvirus thymidine kinase promoter and constitutively expresses low levels of renilla luciferase. Transfected cells were lysed, and the luciferase activities in the cell lysates were analyzed using the Dual Luciferase Reporter assay kit (Promega). Luciferase activity was measured as relative light units (RLUs) using a luminometer (Turner BioSystems, Inc. Sunnyvale, CA, USA).

### Chromatin immunoprecipitation (ChIP) assay

PC12 cells were treated with or without Icariin for 24 hours and used for ChIP analysis as reported [53]. Cells were cross-linked with 1% formaldehyde, and lysed with SDS lysis buffer. Cell lysates were sonicated, and 5% of cell lysate was removed and used to determine the total amount of target DNA in input. Remaining cell lysates were diluted in ChIP dilution buffer. Immunoprecipitation was performed with either NEF2L1 or Xbp-1 antibodies (4 μg) at 4°C overnight. Immune complexes were then mixed with salmon sperm DNA/protein agarose 50% slurry at 4°C for 1 h. After immunoprecipitates were washed sequentially with low salt buffer, high salt buffer, LiCl wash buffer, and Tris EDTA, DNA-protein complexes were eluted with elution buffer, and cross-linking was reversed. Genomic DNA was extracted using phenol/chloroform, and ethanol-precipitated DNA was re-suspended in Tris EDTA and used for the real-time PCR analysis.

### ER stress-induced cell death

PC12 cells were treated with 10 μM tunicamycin (EMD, San Diego, CA, USA) for 24 hours with or without addition of Icariin. Treated cells were collected and stained with FITC-conjugated Annexin V as described [54]. Cell apoptosis was analyzed by flow cytometry to detect expression of Annexin V (eBioscience, San Diego, CA, USA).

## Results

### Icariin induces Synoviolin expression in neuroblastic cells

**Fig 1:**
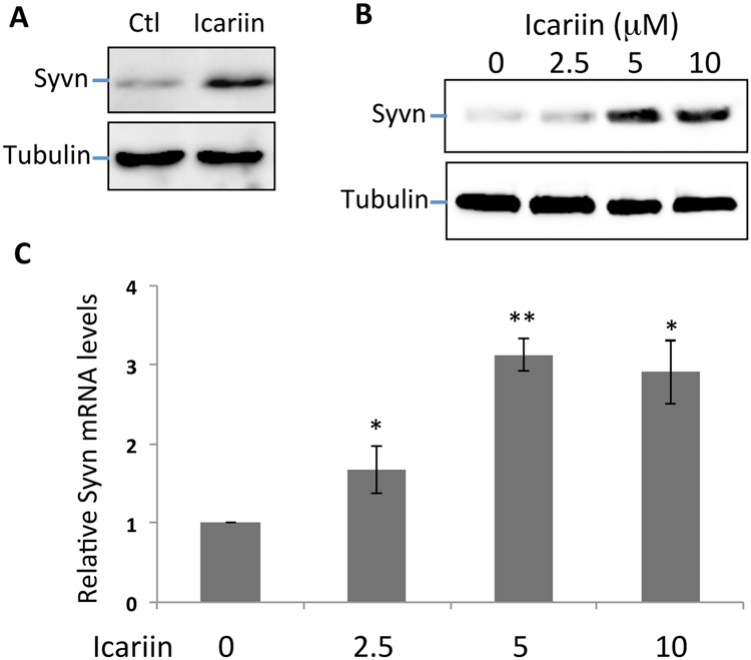
Icariin induces Synoviolin expression at the transcriptional level. PC12 cells were cultured with or without Icariin at different concentrations as indicated. **(A & B)** 24 hours after Icariin treatment, protein expression levels of Synoviolin (top panels) were determined by immunoblotting using β-Actin as a loading control (bottom panels). **(C)** Total RNA was isolated from PC12 cells 24 hours after treatment with or without Icariin. The levels of Synoviolin mRNA were determined by real-time qPCR using GAPDH as an internal control.

We previously discovered that Icariin suppresses neuronal cell death and protects mice against neuronal degenerative disease [10]. To investigate the underlying molecular mechanisms, we analyzed the effects of Icariin on the protein expression of Synoviolin, an anti-apoptotic E3 ubiquitin ligase. PC12 cells were cultured in the presence of Icariin for 24 hours, the treated cells were collected and lysed, and protein expression levels of Synoviolin were examined by immunoblotting. As shown in Fig. 1A, Synoviolin protein expression dramatically increased in PC12 cells after treatment with 5 μM Icariin, indicating that Icariin induces Synoviolin expression in neuronal PC12 cells. We further demonstrated that Icariin treatment enhanced Synoviolin expression in a dose-dependent manner (Fig. 1B)

Next we asked whether Icariin induces Synoviolin expression at the transcriptional level. Total RNA was extracted from Icariin-treated and untreated control PC12 cells. mRNA levels of Synoviolin were determined by real-time qPCR using β-actin mRNA as an internal control. As expected, Synoviolin mRNA levels were significantly higher in the Icariin-treated cells compared to controls (Fig. 1C). Similar to our immunoblot analysis, Icariin induced Synoviolin mRNA expression in a dose-dependent manner. These results indicate that Icariin induces Synoviolin expression at the mRNA level.

### Icariin enhances Synoviolin reporter activity in PC12 cells

**Fig 2:**
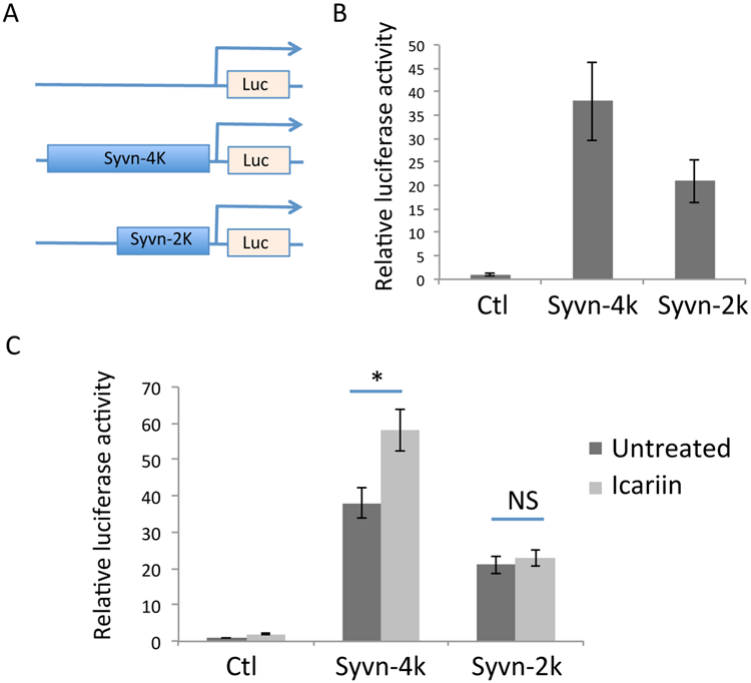
The effects of Icariin on Synoviolin reporter activity. The Synoviolin promoter region was amplified by PCR using genomic DNA from PC12 cells as template. The amplified DNA fragment was subcloned into a luciferase reporter vector. **(A)** Luciferase reporter plasmids carrying the Synoviolin promoter region (2kb and 4kb) or empty vector control plasmid were transfected into PC12 cells. Two days after transfection, luciferase activity was determined. **(B)** 24 hours after transfection, cells were treated with or without Icariin for an additional 24 hours and luciferase activity was determined.

To elucidate the molecular mechanisms underlying Icariin-induced Synoviolin mRNA expression, we subcloned the promoter region of Synoviolin into a luciferase vector (Fig. 2A). We then used the Synoviolin reporter plasmid to test whether Icariin induces Synoviolin expression at the transcriptional level. High levels of luciferase activity were detected in PC12 cells when transfected with Synoviolin reporter plasmids carrying either 2kb or 4kb of the Synoviolin promoter, indicating that this region carries the regulatory elements required for Synoviolin gene transcription. We also detected a significantly higher luciferase activity when the 4kb Synoviolin promoter region reporter was transfected into PC12 cells compared to the 2kb promoter region reporter, suggesting that the distal 2kb region is important for Synoviolin gene transcription. When the transfected cells were treated with Icariin, a significant increase in luciferase activity was detected in cells transfected with the Synoviolin 4kb, but not 2kb, reporter, suggesting that the distal 2kb promoter region also carries the element that is positively regulated by Icariin.

### NFE2L1 binding sites are involved in Icariin-mediated Synoviolin expression

**Fig 3:**
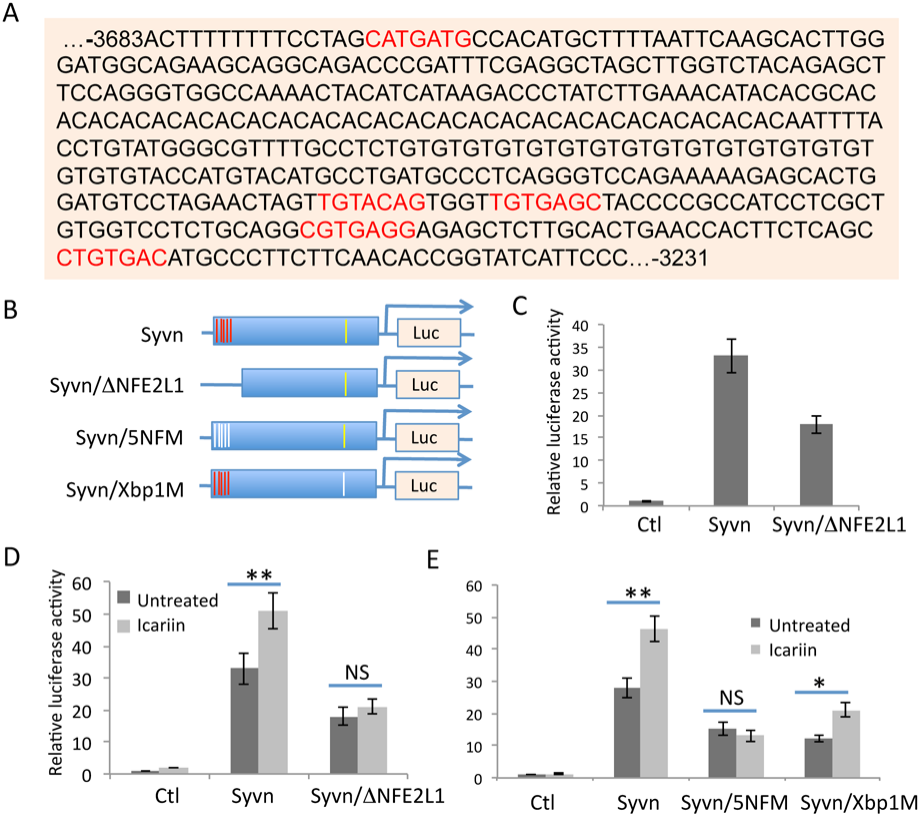
Icariin stimulates Synoviolin reporter activity through NFE2L1 binding sites. **(A)** The distal region that carries the five NFE2L1 transcription factor-binding sites (red) are shown. **(B)** The 4-Kb Synoviolin promoter were cloned into a luciferase reporter plasmid. The NFE2L1- (red lines) and Xbp-1- binding site (yellow line) are indicated. The deletion (second) or point mutation of all the five NFE2L1 (3^rd^ panel) or the Xbp-1 (bottom panel) are illustrated. **(C)** Luciferase reporter plasmids containing the 4kb Synoviolin promoter or a mutant with the four NFE2L1 binding sites deleted (Syvn/ΔNFE2L1) were transfected into PC12 cells. 48 hours after transfection, luciferase activity in the lysates of transfected cells was determined. **(D & E)** The Synoviolin luciferase reporter plasmids or each of the mutants in (B) were transfected into PC12 cells. One day after transfection, cells were treated with Icariin for an additional 24 hours. Cells were then collected and lysed and luciferase activity was determined. Error bars represent data from 5 independent experiments (Mean ± SD). Student’s *t* test was used for the statistical analysis. NS: not significant, *p<0.05 and **p<0.01.

Analysis of the transcription factor binding sites in the 4kb Synoviolin promoter identified one Xbp-1 binding site within the proximal 2kb region and five NFE2L1 binding sites in the distal 3.5–4kb region (Fig. 3A & B). We speculated that the NFE2L1 binding sites are involved in Icariin-mediated Synoviolin gene transcription. To test this hypothesis, the distal promoter region containing the five NFE2L1 binding sites was deleted and luciferase reporter activity was determined (Fig. 3B). As shown in Fig. 3C, deletion of the NFE2L1-binding site region significantly reduced Synoviolin reporter activity, indicating that NFE2L1 is a transcription factor that acts on the Synoviolin promoter in PC12 cells. Interestingly, deletion of the NFE2L1-binding sites in the Synoviolin promoter completely abolished Icariin-stimulated Synoviolin reporter activity (Fig. 3D). To confirm these observation, we generated point mutations of each binding sites (Fig. 3B). As shown in Fig. 3E, mutation of all five NFE2L1-binding sites dramatically inhibited Synoviolin transcription activity. Notably, consistent to our results using the deletion mutate, icariin failed to induce Synoviolin transcription when all the five NFE2L1-binding sites were mutated. In contrast, mutation of the Xbp-1 binding sites, while inhibited the basal level of Synoviolin transcription activity, did not affect icariin-induced Synoviolin expression (Fig. 3E). These results suggest NFE2L1 is responsible for Icariin-induced Synoviolin expression.

### Icariin induces Synoviolin gene transcription in an NFE2L1-dependent manner

**Fig 4:**
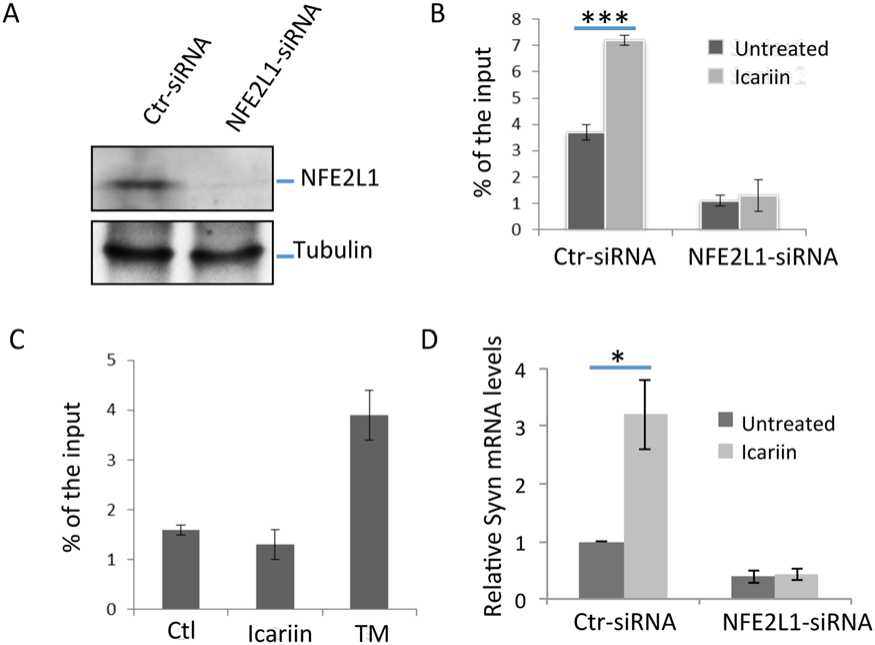
NFE2L1 knockdown abolishes Icariin-induced Synoviolin expression. **(A)** PC12 cells were transfected with NFE2L1 shRNA or control shRNA. Two days after transfection, the expression levels of NFE2L1 were analyzed by immunoblotting using anti-NFE2L1 Abs (top panel). The expression levels of tubulin protein were determined as a loading control (bottom panel). **(B & C)** PC12 or its NFE2L1 knockdown cells were treated with or without Icariin for 24 hours. ChIP assay using anti-NFE2L1 (B) or with anti-Xbp-1 (C) antibodies. Tunicamycin (TM) treated cells were used as a positive control for Xbp-1 ChIP analysis. **(D)** The Synoviolin luciferase reporter plasmids were co-transfected with NFE2L1-shRNA or control shRNA plasmid into PC12 cells. One day after transfection, cells were treated with or without Icariin for an additional 24 hours. The luciferase activity in the lysates of transfected cells was determined. Error bars represent data from 5 independent experiments (Mean ± SD). Student’s *t* test was used for the statistical analysis. **p<0.01, and ***p<0.001.

We then used an shRNA-mediated knockdown approach to test our hypothesis that Icariin promotes Synoviolin gene expression through NFE2L1. Immunoblotting analysis confirmed that a specific shRNA efficiently suppressed NFE2L1 protein expression in PC12 cells (Fig. 4A). ChIP analysis detected a direct binding of the NFE2L1 to the distal region of Synoviolin promoter, which was largely diminished by NFE2L1 knockdown. Notably, Icariin treatment significantly increased NFE2L1 binding to Synoviolin promoter in PC12 cells (Fig. 4B). In contrast, Xbp-1 binding to Synoviolin promoter was not affected by Icariin treatment (Fig. 4C). In addition, we detected an approximate 60% reduction in Synoviolin mRNA levels in PC12 cells with NFE2L1 knockdown compared to control shRNA transfected cells. Interestingly, in contrast to the average 3-fold increase in Synoviolin mRNA expression seen upon Icariin treatment of control shRNA transfected PC12 cells, Icariin failed to enhance Synoviolin mRNA transcription in PC12 cells when NFE2L1 was knocked down. These results clearly indicate that Icariin enhances Synoviolin expression in an NFE2L1-dependent manner.

### Icariin protects against neuronal cell death through upregulation of Synoviolin

**Fig 5:**
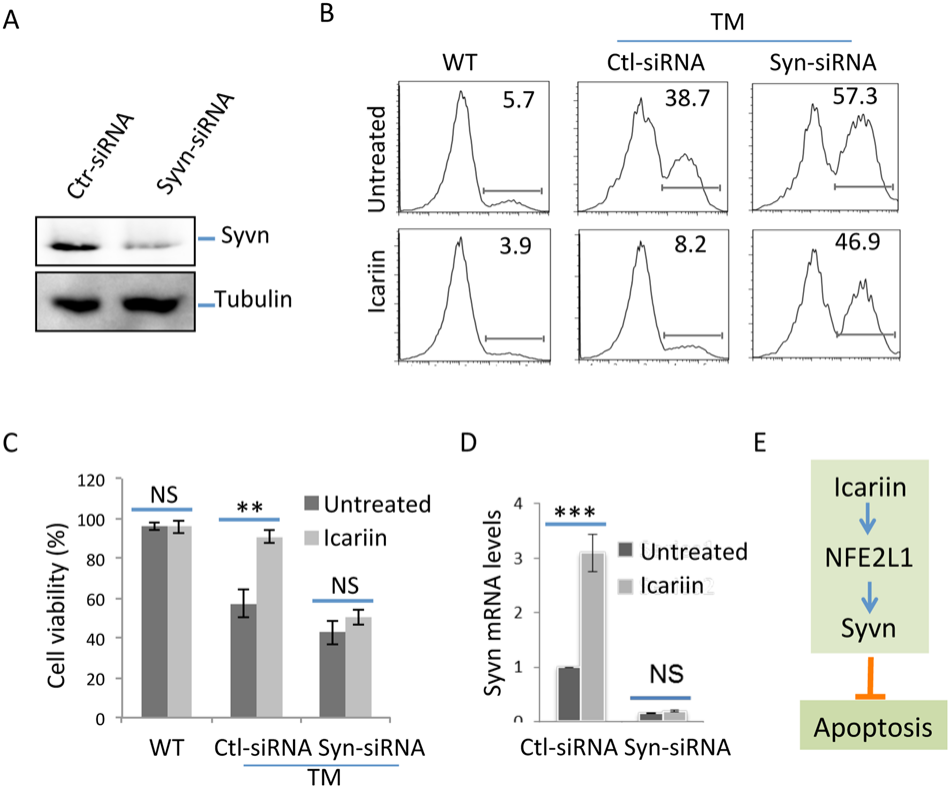
Icariin protects ER stress-induced PC12 cell death through Synoviolin. **(A)** PC12 cells were transfected with Synoviolin shRNA or control shRNA. Two days after transfection, the expression levels of Synoviolin were analyzed by immunoblotting using anti-Synoviolin Abs (top panel). The expression levels of tubulin protein were determined as a loading control (bottom panel). **(B & C)** ER stress-induced cell death by tunicamycin treatment in wild type control and Synoviolin knockdown cells were analyzed by flow cytometry for the expression of annexin V. Representative images from 5 independent experiments are shown (B). Viability of PC12 cells from 5 independent experiments is shown (Mean ± SD). Student’s *t* test was used for the statistical analysis. **p< 0.05. NS: not significant. **(D)** The mRNA expression levels of Synoviolin in WT and Synoviolin knockdown PC12 cells after Icariin treatment was confirmed by real-time PCR. **(E)** A proposed model of Icariin in protecting PC12 cells against apoptosis. Error bars represent data from 5 independent experiments (Mean ± SD). Student’s *t* test was used for the statistical analysis. **p<0.01, and ***p<0.001.

We and others have shown that Icariin holds great therapeutic potential, with evidence of protection against neuronal degenerative disease in mice. One of the proposed underlying mechanisms is that Icariin protects neuronal cells from apoptosis [9, 10]. Since Synoviolin is an anti-apoptotic factor, our discovery that Icariin induces Synoviolin gene transcription suggested that Icariin may inhibit neuronal cell death through upregulation of Synoviolin. To test this hypothesis, we used an shRNA-mediated gene knockdown approach to inhibit Synoviolin expression and test whether Icariin is able to protect PC12 cells from ER stress-induced apoptosis in the absence of Synoviolin. Synoviolin-specific shRNA largely inhibited Synoviolin expression in PC12 cells as determined by immunoblotting (Fig. 5A). As previously reported, treatment of PC12 cells with the pharmacological ER stress inducer tunicamycin at 10 μg/ml resulted in an average of 30% annexin V-positive apoptotic cells. Importantly, treatment of PC12 cells with Icariin fully protected against tunicamycin-induced cell death (Fig. 5B). As expected, tunicamycin treatment of PC12 cells with Synoviolin knocked down led to an elevated percentage of apoptotic cells, from 30% to approximately 50%. Notably, addition of Icariin failed to protect Synoviolin knockdown PC12 cells from tunicamycin-induced apoptosis (Fig. 5B & 5C), indicating that Synoviolin function is essential for Icariin-mediated protection of neuronal cells from ER stress-induced apoptosis. Real-time PCR analysis confirmed that Synoviolin expression was sufficiently inhibited by siRNA even after Icariin treatment (Fig. 5D). Therefore, our study found that Synoviolin upregulation plays a critical role in the protective function of Icariin.

Based on our discoveries, we proposed a model for Icariin in protecting against neuronal cell death (Fig. 5E): Icariin induces Synoviolin expression in PC12 cells through the transcription factor NFE2L1. Upregulated Synoviolin then inhibits PC12 cell apoptosis when the ER stress response occurs.

## Discussion

The therapeutic potential of Icariin for the treatment of neuronal degenerative disease has stimulated extensive research into the molecular mechanisms underlying the protective effect of Icariin against apoptosis of neuronal cells. Here, we demonstrated that Icariin protects ER stress-induced PC12 cell death through induction of Synoviolin expression. This conclusion is supported by the following observations: first, Icariin induced both protein and mRNA expression of Synoviolin in PC12 cells; second, the transcription factor NFE2L1 was required for Icariin-mediated Synoviolin promoter activity, with knockdown of NFE2L1 expression largely abolishing Icariin-induced Synoviolin expression; and third, suppression of Synoviolin expression by shRNA-mediated knockdown abolished the ability of Icariin to protect PC12 cells from ER stress-induced cell death.

Synoviolin is a known anti-apoptotic factor during ER stress-induced cell death. A significant reduction in Synoviolin protein levels has been detected in the cerebral cortex of Alzheimer’s disease patients [55]. However, the mediators of Synoviolin transcriptional regulation in neurons remain unclear. Our observations here indicate that the transcription factor NFE2L1 is involved in regulating Synoviolin mRNA transcription in PC12 cells, providing a possible explanation for the reduced Synoviolin expression in the brains of AD patients. Notably, Lee et al discovered that mice with a late-stage deletion of NFE2L1 in neuronal cells have dysregulated proteasome gene expression and develop neurodegeneration syndromes [56]. It will be interesting to study the Synoviolin promoter-binding activity of NFE2L1 in neurons from AD patients. Several studies have shown that Synoviolin suppresses neuronal cell death in human neuronal cells and experimental rodent models of neuronal degenerative disease [47, 55, 57–69]. Therefore, our discovery that Icariin induces Synoviolin expression to suppress ER stress-induced neuron cell apoptosis provides a rationale for the clinical application of Icariin in treatment of patients with neuronal degenerative diseases, particularly in patients with reduced Synoviolin expression levels.

There are at least five conserved NFE2L1-specific binding sites on the distal region of the mouse, rat, and human Synoviolin promoter. These NFE2L1-binding sites are absolutely required for Icariin activity in promoting Synoviolin mRNA transcription, as deletion of the five NFE2L1-binding sites fully abolished Icariin-induced Synoviolin reporter activity. NFE2L1 has been identified as a substrate of Synoviolin-mediated ubiquitination and degradation [70, 71]. Therefore, our finding that NFE2L1 is a transcription factor acting on the Synoviolin promoter implies that a negative feedback loop regulates Synoviolin expression. Similarly, the X box protein 1 (Xbp-1) is another transcription factor involved in Synoviolin gene expression [21, 72], and we identified an Xbp-1 binding site in the promoter region of Synoviolin locus. We have previously shown that IRE1α, an ER stress transducer and the only known factor upstream of the Xbp-1 activator that acts by splicing Xbp-1 mRNA, is ubiquitinated and degraded by Synoviolin [48]. A similar negative regulatory feedback loop between Synoviolin and Xbp-1 may also exist. Further studies are needed to explore how these two feedback loops are regulated and whether they occur in different physiological and pathological settings in neurons. In addition to NFE2L1 and Xbp-1, Ets-1 binding sites (EBS-1) have been identified and the Ets-1-mediated Synoviolin expression plays important roles in cellular hemostasis [73]. Moreover, Izumi et al demonstrated that the interleukin enhancer binding factor 3 (ILF-3) activates the Synoviolin promoter via association with GABP-α in rheumatoid synovial cells during joint inflammation [74]. Therefore, Synoviolin gene appears to be regulated by multiple transcription factors.

Our study shows that a full protection against ER stress-induced PC12 cell death requires Synoviolin expression. It has been well established that Synoviolin is a critical anti-apoptotic factor that suppresses ER stress-induced cell death by directly degrading misfolded proteins through the ubiquitin pathway [75]. Therefore, together with the fact that the ER stress response is often associated with the pathogenesis of neuronal degenerative diseases in humans [35–42], our study suggests that Icariin may be particularly effective in the treatment of neuronal degenerative diseases with elevated ER stress response.

## Supporting Information

S1 Fig Values of the graph figures in the study. The averages values and their standard deviations (mean ± SD) are indicated for the figures including Fig. 1C, 2B & C, 3C-E, 4B-D and 5C & D.
(PDF)
