# Supplementary figures and images for: Icariin Induces Synoviolin Expression through NFE2L1 to Protect Neurons from ER Stress-Induced Apoptosis
Source: PLoS One. 2015 Mar 25;10(3):e0119955. doi: 10.1371/journal.pone.0119955 (PMC4373914; doi:10.1371/journal.pone.0119955)

Fig. 1C

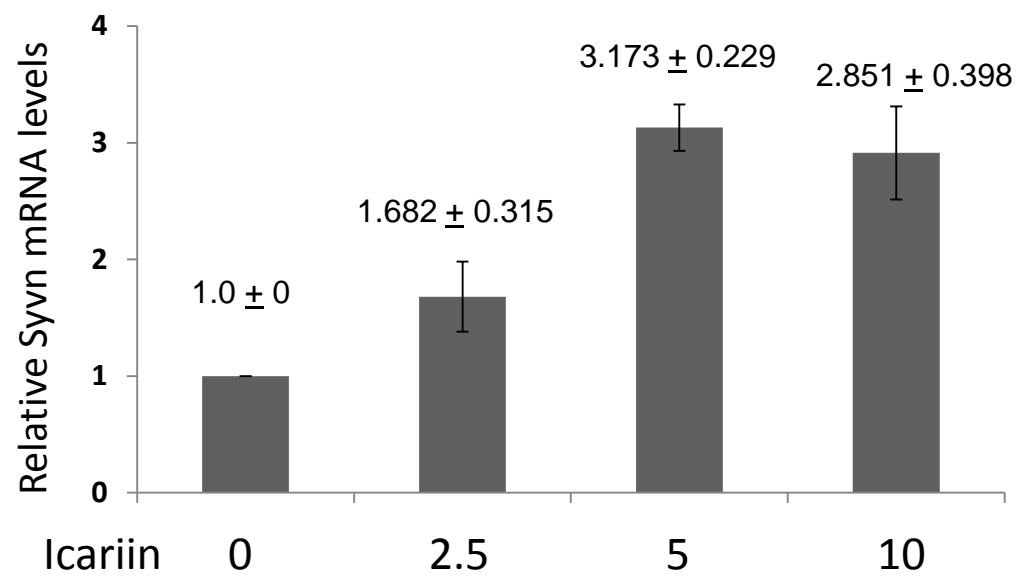

Fig.2B

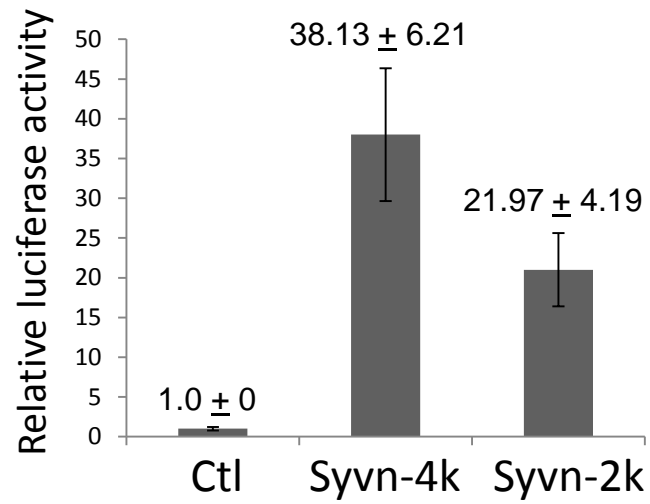

Fig. 2C

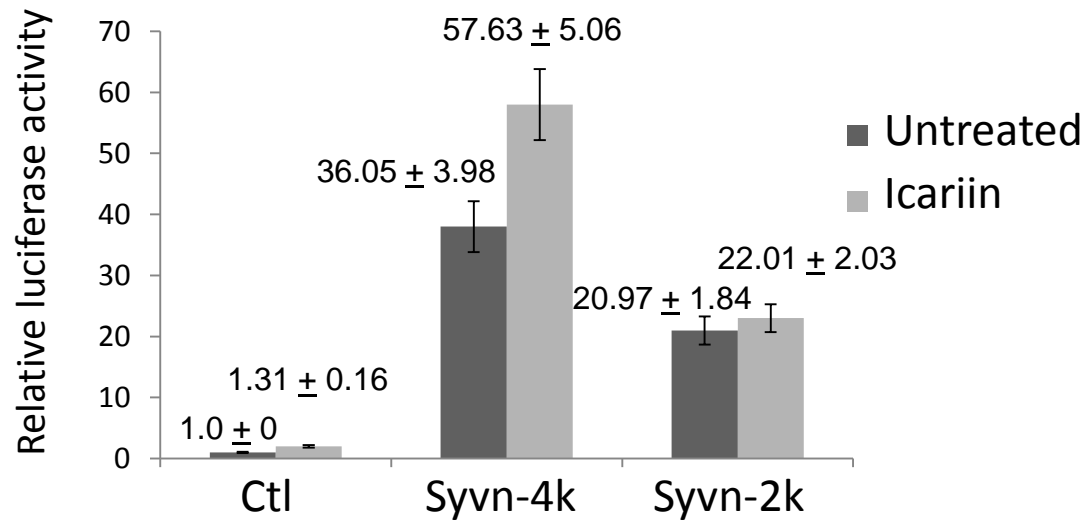

Fig.3C

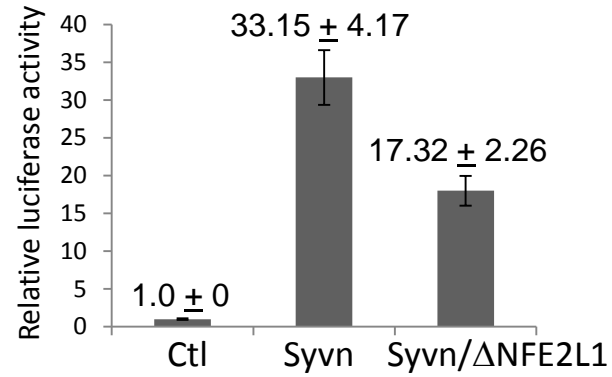

Fig. 3D

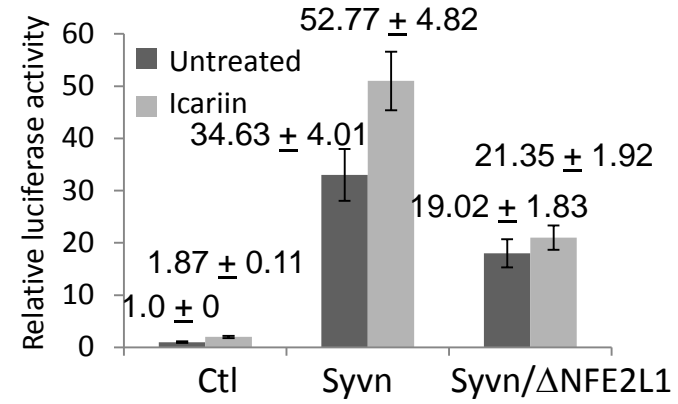

Fig.3E

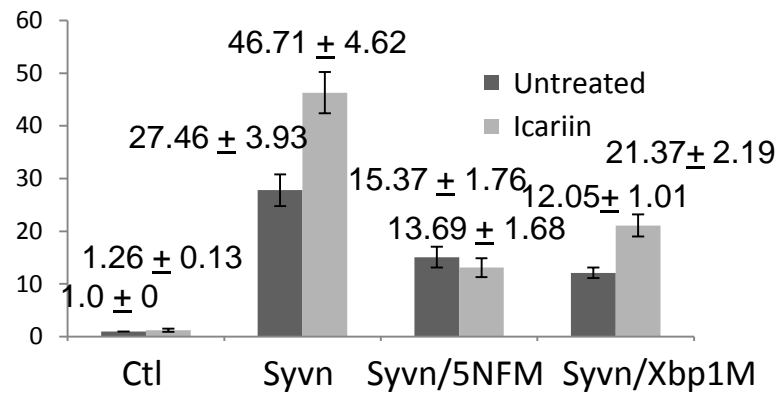

Fig. 4B

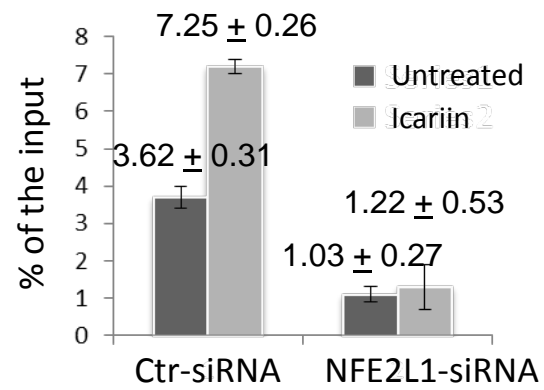

Fi. 4C

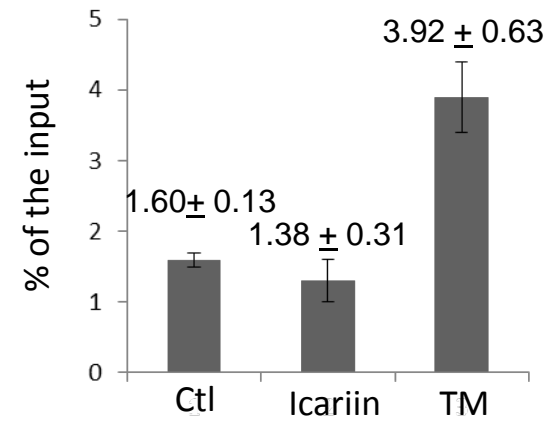

Fig. 4D

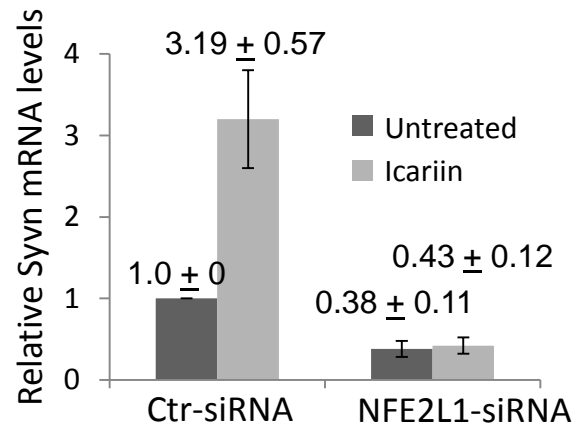

Fig. 5C

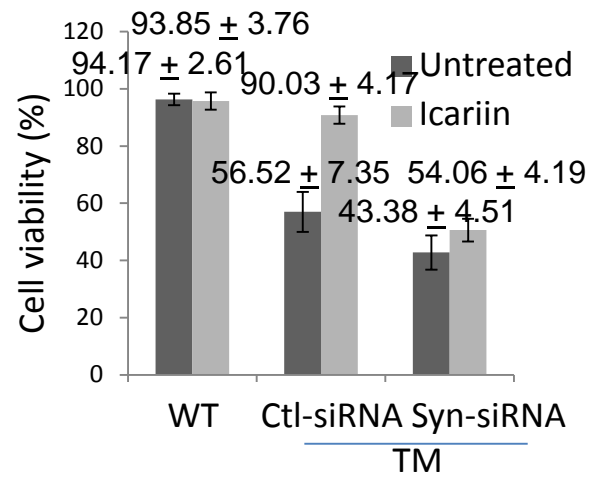

Fig.5D

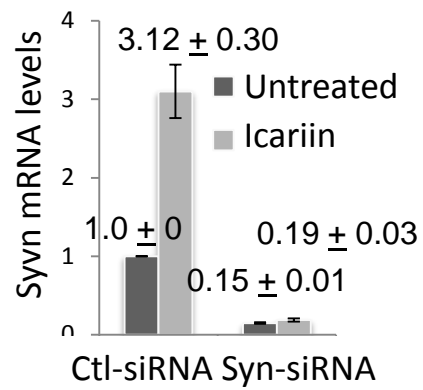

Supplement: S1 Fig — The averages values and their standard deviations (mean ± SD) are indicated for the figures including Fig. 1C, 2B & C, 3C-E, 4B-D and 5C & D. (PDF) [file pone.0119955.s001.pdf]
